# Assessment of different strategies for scalable production and proliferation of human myoblasts

**DOI:** 10.1111/cpr.12602

**Published:** 2019-03-19

**Authors:** Min‐Wen Jason Chua, Ege Deniz Yildirim, Jun‐Hao Elwin Tan, Yan‐Jiang Benjamin Chua, Suet‐Mei Crystal Low, Suet Lee Shirley Ding, Chun‐wei Li, Zongmin Jiang, Bin Tean Teh, Kang Yu, Ng Shyh‐Chang

**Affiliations:** ^1^ NUS Graduate School for Integrative Sciences and Engineering National University of Singapore Singapore City Singapore; ^2^ Stem Cell & Regenerative Biology Genome Institute of Singapore, Agency for Science Technology and Research Singapore City Singapore; ^3^ Laboratory of Cancer Therapeutics, Program in Cancer and Stem Cell Biology Duke‐NUS Medical School Singapore City Singapore; ^4^ Institute of Molecular and Cell Biology Agency for Science Technology and Research Singapore City Singapore; ^5^ Division of Medical Science, Laboratory of Cancer Epigenome National Cancer Centre Singapore Singapore City Singapore; ^6^ Department of Biochemistry, Yong Loo Lin School of Medicine National University of Singapore Singapore City Singapore; ^7^ Department of Clinical Nutrition, Peking Union Medical College Hospital Chinese Academy of Medical Sciences & Peking Union Medical College Beijing China; ^8^ State Key Laboratory of Stem Cell and Reproductive Biology, Institute of Zoology Chinese Academy of Sciences Beijing China; ^9^ University of Chinese Academy of Sciences Beijing China; ^10^ Institute of Stem cell and Regeneration Chinese Academy of Sciences Beijing China

**Keywords:** adult stem cells, cell fate, cellular differentiation, embryonic stem cells, immortalization, musculoskeletal system

## Abstract

**Objectives:**

Myoblast transfer therapy (MTT) is a technique to replace muscle satellite cells with genetically repaired or healthy myoblasts, to treat muscular dystrophies. However, clinical trials with human myoblasts were ineffective, showing almost no benefit with MTT. One important obstacle is the rapid senescence of human myoblasts. The main purpose of our study was to compare the various methods for scalable generation of proliferative human myoblasts.

**Methods:**

We compared the immortalization of primary myoblasts with hTERT, cyclin D1 and CDK4^R24C^, two chemically defined methods for deriving myoblasts from pluripotent human embryonic stem cells (hESCs), and introduction of viral MyoD into hESC‐myoblasts.

**Results:**

Our results show that, while all the strategies above are suboptimal at generating bona fide human myoblasts that can both proliferate and differentiate robustly, chemically defined hESC‐monolayer‐myoblasts show the most promise in differentiation potential.

**Conclusions:**

Further efforts to optimize the chemically defined differentiation of hESC‐monolayer‐myoblasts would be the most promising strategy for the scalable generation of human myoblasts, for applications in MTT and high‐throughput drug screening.

## 1 INTRODUCTION

Skeletal muscle stem cells exist as satellite cells in adult mammalian skeletal muscles. These stem cells are located on the periphery of myofibers’ plasma membrane, beneath the muscle basal lamina or endomysium. Postnatal growth, maintenance and regeneration of skeletal muscles in vivo rely on muscle satellite cells that proliferate as myoblasts, which are marked by expression of PAX7 and MyoD but not myogenin (MYOG).^1^ In the early stages of myoblast differentiation, MyoD+ MYOG+ myocytes begin to accumulate muscle‐specific α–actin 1 (ACTA1) and other myofilament components such as embryonic myosin heavy chain (MYH3).^1^ Subsequently, myocytes can fuse together to form α–actinin+ MYOG+ multinucleated myotubes and finally myofibers, which are marked by very high levels of mature myofilament components such as adult myosin heavy chain isoforms.^1^ Phenotypic analyses of genetic mouse models strongly suggest that the loss of muscle satellite cells abolishes the regenerative capacity of adult skeletal muscles.^1^, ^2^ Dysfunction in the proliferative muscle satellite cells, or myoblasts, leads to a decrease in regenerative capacity of muscles, resulting in muscle dysfunction during both normal ageing and the progression of muscle degenerative diseases, such as muscular dystrophies.

Amongst the large variety of heritable muscular dystrophies, Duchenne muscular dystrophy (DMD) is the most common, affecting one in 3600 boys due to a mutation in the dystrophin gene.^3^, ^4^ Mouse models bearing mutations similar to those described in human muscular dystrophies, such as the dystrophin mutation in DMD, have been employed to develop myoblast transfer therapy (MTT) against muscular dystrophies. This is a technique to replace muscle satellite cells with genetically repaired or healthy myoblasts, to treat the muscular dystrophy.^5^, ^6^ However, clinical trials with human myoblasts were ineffective, showing almost no benefit with MTT.^7^, ^8^, ^9^ In addition to obstacles such as the limited migration capabilities of human myoblasts, and the immune response during MTT, another important obstacle is the rapid senescence of human myoblasts. Unlike primary rodent myoblasts, primary human myoblasts rapidly show senescence in vitro. This limitation is manifested as progressively compromised differentiation and proliferation potential, during in vitro culture.^10^, ^11^ This limitation not only prevents us from achieving MTT for muscular dystrophy patients, but also limits our ability to conduct high‐throughput drug screening and carry out molecular characterization in human myoblasts with high reproducibility.^12^

To overcome this limitation, several approaches have been used, such as expression of the simian virus 40 large T (SV40‐LT) antigen and human telomerase reverse transcriptase (hTERT).^13^, ^14^ SV40‐LT is an oncogenic protein that forcibly promotes cell cycle turnover, but its expression can cause genomic instability and disrupt myogenesis.^13^ By combining lentiviral hTERT, with cyclin D1, and/or oncogenic CDK4^R24C^, human myoblasts could proliferate indefinitely while maintaining a normal karyotype.^12^, ^15^ However, the immortalized human myoblasts could also undergo osteogenesis and adipogenesis under appropriate conditions,^15^ a phenomenon that is never seen in primary human myoblasts, suggesting that immortalization had deranged their differentiation potential.

An alternative strategy to generate human myoblasts in large scale is by directed differentiation of human embryonic stem cells (hESCs), to recapitulate development to form cell lineages that are similar to their in vivo counterparts. Directed differentiation of hESCs to specific lineages for cell therapies is showing promise in clinical settings and in preclinical animal models for various diseases.^16^ However, for many cell lineages, directed differentiation results in progeny that are heterogeneous and functionally immature compared to primary in vivo cells.^17^, ^18^ Protocols that are used to differentiate skeletal muscle cells from hESCs often require virus‐mediated overexpression of transcription factor transgenes.^19^, ^20^, ^21^, ^22^ Although many transgene‐free, chemically defined protocols for generating myoblasts from hESCs have also been described,^23^, ^24^, ^25^, ^26^ their heterogeneity and differentiation potential remain poorly characterized in comparison with other methods.

Here, we compared the various methods for generating human myoblasts at large scale, including immortalization of primary myoblasts with hTERT, CDK4^R24C^, cyclin D1,^15^ two chemically defined methods for hESC‐myoblasts,^23^, ^26^ and introduction of viral MyoD into hESC‐myoblasts. Our results show that all the methods above are suboptimal at generating bona fide human myoblasts that can both proliferate and differentiate robustly. Our results further suggest that hESC‐myoblasts show more promise in differentiation potential, and that further efforts to optimize the directed differentiation of hESC‐myoblasts would be useful.

## 2 METHODS

### 2.1 Cell culture and virus production

The female (WA07) hESC line and the male (WA01) hESC line from WiCell, certified to be mycoplasma‐free and bona fide human pluripotent stem cells, were propagated in mTeSR1 (Stem Cell Technologies) supplemented with 1% penicillin‐streptomycin (Gibco) and were maintained feeder‐free on hESC‐qualified Matrigel (BD Biosciences) in a humidified atmosphere (5% CO_2_, 37°C). The medium was changed daily. Both hESC lines were passaged using collagenase Type IV (Gibco) at a 1:4‐1:6 split ratio every 4‐6 days, and routinely checked every 2 months to prevent any mycoplasma contamination.

Overtly differentiated hESC colonies were mechanically removed prior to induction of differentiation. When the hESC colony density on the plate was approximately 30%‐40%, differentiation of hESCs was induced. For EB differentiation into myoblasts and myotubes, we exactly followed the protocol of Xu et al.^23^ For monolayer differentiation into myoblasts and myotubes, we exactly followed the protocol of Shelton et al.^25^, ^26^ All culture media were refreshed daily throughout the protocols.

Commercial primary adult HSKM myoblasts were derived from healthy adult patient donors (Gibco), and cultured in growth medium composed of DMEM/F12 supplemented with 10% heat‐inactivated foetal bovine serum (FBS) (GE), 1% penicillin‐streptomycin (Gibco) and 1% l‐glutamine (Gibco) in a humidified atmosphere (5% CO_2_, 37°C) for <5 passages. About 100% confluent HSKM myoblasts were induced to differentiate into myotubes under growth factor withdrawal conditions with 2% horse serum in DMEM supplemented with 1% penicillin‐streptomycin (Gibco) and 1% l‐glutamine (Gibco) for 7 days.

GP2‐293 cells (Clontech) were seeded at 10% confluency and transfected with a 12 µL : 3.33 µg : 0.66 µg mix of PEI (1 mg/mL) : retroviral plasmids (Addgene #1773, #26357) : VSV‐G envelope plasmid (Addgene #8454). 293FT HEK cells (Clontech) were seeded at 10% confluency and transfected with a 42 µL:7 µg:6.3 µg:0.7 µg mix of PEI (1 mg/mL): lentiviral plasmid (Addgene #19119): dR8.2 packaging plasmid (Addgene #8455): VSV‐G envelope plasmid (Addgene #8454). 293FT and GP2 cells were initially cultured in DMEM (Gibco) with 10% FBS (GE Healthcare), 1% l‐glutamine (Gibco) and 1% penicillin‐streptomycin (Gibco). 24‐hour post‐transfection, growth medium was replaced with DMEM (Gibco) with 20% FBS (GE), 1% l‐glutamine (Gibco) and 1% penicillin‐streptomycin (Gibco). The following plasmids were used to make the viruses: pBABE‐MDER (gift from Stephen Tapscott; Addgene plasmid #13494), pBABE‐neo‐hTERT (gift from Bob Weinberg; Addgene plasmid #1774), pBABE‐hygro CDK4 R24C (gift from Bob Weinberg; Addgene plasmid #11254) and pBABE puro cyclinD1 HA (gift from William Hahn; Addgene plasmid #9050).

### 2.2 Virus transduction and selection

HSKM myoblasts were seeded in six‐well plates (Falcon) in growth medium comprising of DMEM‐F12 (Gibco) with 20% heat‐inactivated FBS (GE), 1% l‐glutamine (Gibco) and 1% penicillin‐streptomycin (Gibco). Cells were then transduced with 0.1‐1 mL of concentrated viral supernatant in the presence of polybrene (Sigma), and incubated for 16‐24 hours. Transduced cells were selected with growth media containing either hygromycin (0.5 mg/mL) for 6‐8 days, puromycin (1 µg/mL) for 3 days, or G418 (2 mg/mL) for 5‐7 days (InvivoGen).

### 2.3 Population doubling curve

1.5 × 10^4^ cells were seeded in one gelatin‐coated well of a six‐well plate (Falcon) with growth medium comprising of DMEM/F‐12 (Gibco) with 20% heat‐inactivated (FBS; Gibco), 1% l‐glutamine (Gibco) and 1% penicillin‐streptomycin (Gibco). Upon reaching a confluency of 80%‐100%, cells were lifted with 0.25% trypsin (Gibco) and counted, and 1.5 × 10^4^ cells were then subcultured. This process was repeated until cells could no longer achieve 80% confluency, or until a period of 100 days. Recorded cell counts were calculated as cumulative population doubling levels and plotted over the number of days in culture.

### 2.4 Immunofluorescence

Cells were first washed with PBS (Thermo Fisher) and fixed with 4% PFA (MS). Cells were stained with the following primary antibodies and concentrations, Desmin (ab6322; Abcam; 1:250), PAX7 (Pax7‐c; DSHB; 5 μg/mL), MYOD1 (sc‐760; Santa Cruz; 1:50), MHC‐Alexa Fluor 488 (53‐6503‐82 [MF‐20]; Thermo Fisher; 1:100), α‐actinin (A7811; Sigma; 1:500) and myogenin (sc‐576; Santa Cruz; 1:200). The following secondary antibodies were also used together with non‐conjugated primary antibodies, Goat‐anti‐mouse Alexa Fluor 488 (A11001; Thermo Fisher; 1:500), Goat‐anti‐rabbit Alexa Fluor 594 (A11012; Thermo Fisher; 1:500) and Goat‐anti‐mouse Alexa Fluor 647 (A21235; Thermo Fisher; 1:500). DAPI (d9542; Sigma) was used as a nuclear counter stain according to manufacturer’s recommendations. Stained cells were imaged with a Zeiss fluorescence microscope.

### 2.5 Quantitative PCR

RNA was extracted by TRIzol (Thermo Fisher) and reverse transcribed with Superscript III (Thermo Fisher) according to manufacturer’s instructions. The resulting cDNA was diluted 5× before performing qPCR with KAPA SYBR FAST on ABI Prism 7900HT (Applied Biosystems) according to manufacturers’ instructions. For primer sequences, see Table 1.

**Table 1:** List of qPCR primers for conventional 384‐well plate qPCR

Gene	Orientation	Sequence 5′‐3′
PAX3	FORWARD	CTC CAC GCT CCG GAT AGT TC
	REVERSE	ATC TTG TGG CGG ATG TGG TT
PAX7	FORWARD	CGT GCT CAG AAT CAA GTT CG
	REVERSE	GTC AGG TTC CGA CTC CAC AT
ALX4	FORWARD	ATG AAT GCT GAG ACT TGC GTC
	REVERSE	GGG AAA TGC CCT AAA AGG CG
SOX2	FORWARD	TTG TCG GAG ACG GAG AAG CG
	REVERSE	TGA CCA CCG AAC CCA TGG AG
PAX6	FORWARD	TCT AAT CGA AGG GCC AAA TG
	REVERSE	TGT GAG GGC TGT GTC TGT TC
TWIST1	FORWARD	CTG CAG CAC CGG CAC CGT TT
	REVERSE	CCC AAC GGC TGG ACG CAC AC
FLK1	FORWARD	AGT GAT CGG AAA TGA CAC TGG A
	REVERSE	GCA CAA AGT GAC ACG TTG AGA T
AFP	FORWARD	AGC TTG GTG GTG GAT GAA AC
	REVERSE	CCC TCT TCA GCA AAG CAG AC
GATA4	FORWARD	CTA GAC CGT GGG TTT TGC AT
	REVERSE	TGG GTT AAG TGC CCC TGT AG
VECAD	FORWARD	TGT GAT GTT GGC CGT GTT AT
	REVERSE	CAG CCC AAA GTG TGT GAG AA
MYOD1	FORWARD	CGG CAT GAT GGA CTA CAG C
	REVERSE	CAG GCA GTC TAG GCT CGA C
MYOG	FORWARD	GGG GAA AAC TAC CTG CCT G
	REVERSE	AGG CGC TCG ATG TAC TGG A
EN1	FORWARD	GTGGTCAAGACTGACTCACGC
	REVERSE	GCTTGTCTTCCTTCTCGTTCTT
NCAM1	FORWARD	ATG GAA ACT CTA TTA AAG TGA ACC TG
	REVERSE	TAG ACC TCA TAC TCA GCA TTC CAG T
ACTA1	FORWARD	CGA CAT CAG GAA GGA CCT GTA TGC C
	REVERSE	GGC CTC GTC GTA CTC CTG CTT GG
MYHC	FORWARD	TTC ATT GGG GTC TTG GAC AT
	REVERSE	AAC GTC CAC TCA ATG CCT TC
MYH3	FORWARD	ATT GCT TCG TGG TGG ACT CA
	REVERSE	GGC CAT GTC TTC GAT CCT GTC
MYH8	FORWARD	TAA ACA CAC CTG CCT GAT GC
	REVERSE	TCA GCT TTA ACA GGA AAA TAA ACG
MYH7	FORWARD	TGC CAC ATC TTG ATC TGC TC
	REVERSE	CTC GGC TTC AAG GAA AAT TG
MYH2	FORWARD	CTG ATG CCA TGG AAT GAC TG
	REVERSE	CCC TAT GCT TTA TTT CCT TTG C
OCT4	FORWARD	GAC AGG GGG AGG GGA GGA GCT AGG
	REVERSE	CTT CCC TCC AAC CAG TTG CCC CAA AC
GAPDH	FORWARD	TGG TAT CGT GGA AGG ACT CA
	REVERSE	TTC AGC TCA GGG ATG ACC TT

## 3 RESULTS

### 3.1 Immortalization of human myoblasts with hTERT, CDK4^R24C^ and cyclin D1

**Figure 1:**
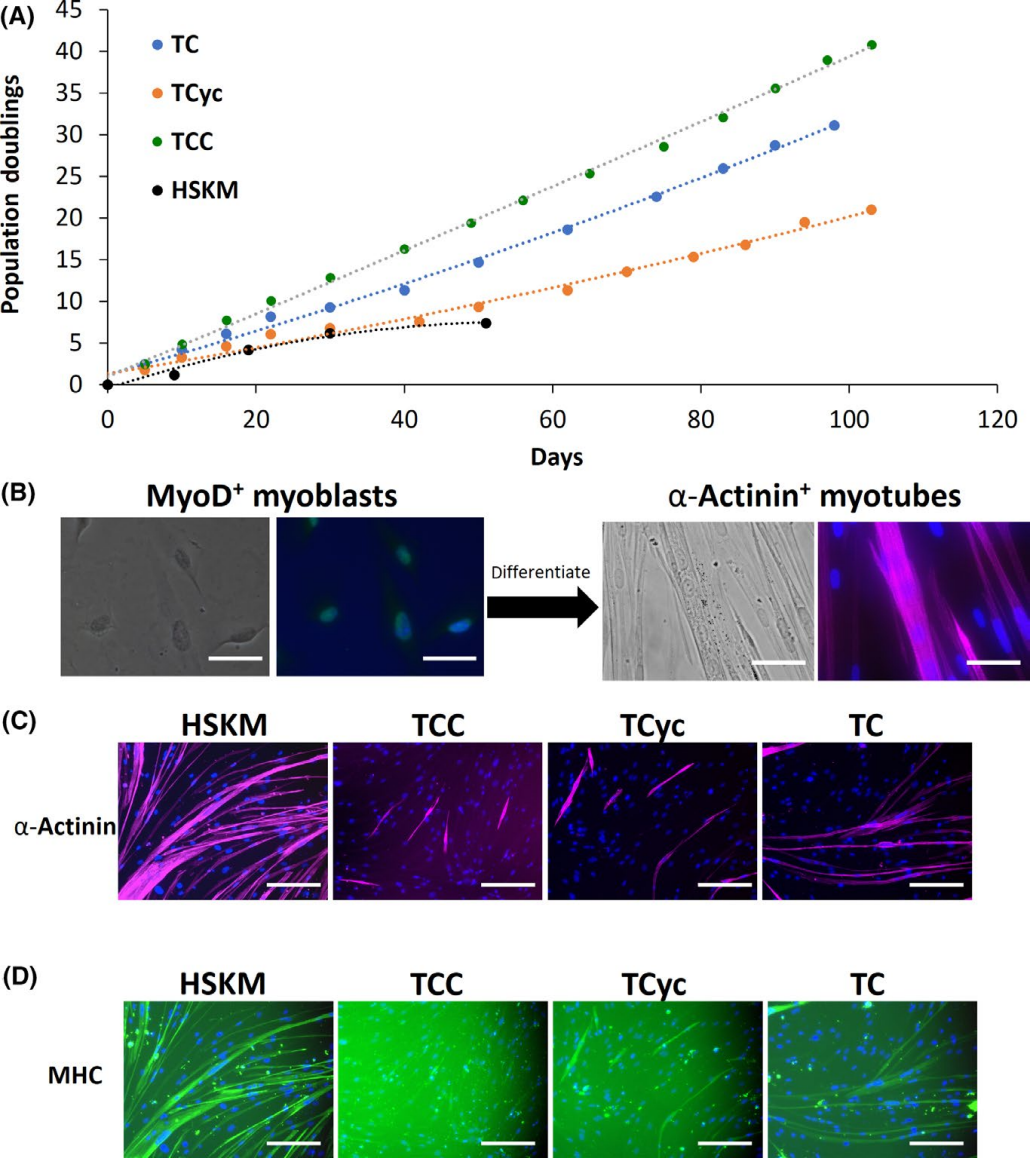
Immortalization of adult primary human skeletal muscle (HSKM) myoblasts. A, Population doubling curves for HSKM myoblasts (black), and HSKM myoblasts transduced with lentiviral hTERT and Cyclin D1 (TCyc, orange), or CDK4^R24C^ (TC, blue), or CDK4^R24C^ and Cyclin D1 (TCC, green). While adult HSKM myoblasts underwent senescence by the 6th population doubling at 30 d, the other cells continued to proliferate steadily for 100 d and beyond. B, High magnification (40×) phase contrast and immunofluorescence images of MyoD+ (green) myoblasts and, after fusion and differentiation, α‐actinin+ (purple) multinucleated myotubes. Cells were counterstained with DAPI to visualize the myonuclei. Scale bars 25 μm. C, Immunofluorescence staining for the myotube marker α‐actinin in HSKM, TCC (hTERT, CDK4R24C, Cyclin D1), hTERT‐Cyclin D1 and hTERT‐CDK4 myoblasts that were subjected to myogenic differentiation. Cells were counterstained with DAPI to visualize the myonuclei. Scale bars 50 μm. D, Immunofluorescence staining for the myotube marker myosin heavy chain (MHC) in HSKM, TCC (hTERT, CDK4^R24C^, Cyclin D1), hTERT‐Cyclin D1 and hTERT‐CDK4 myoblasts that were subjected to myogenic differentiation. Cells were counterstained with DAPI to visualize the myonuclei. Scale bars 50 μm

**Figure 2:**
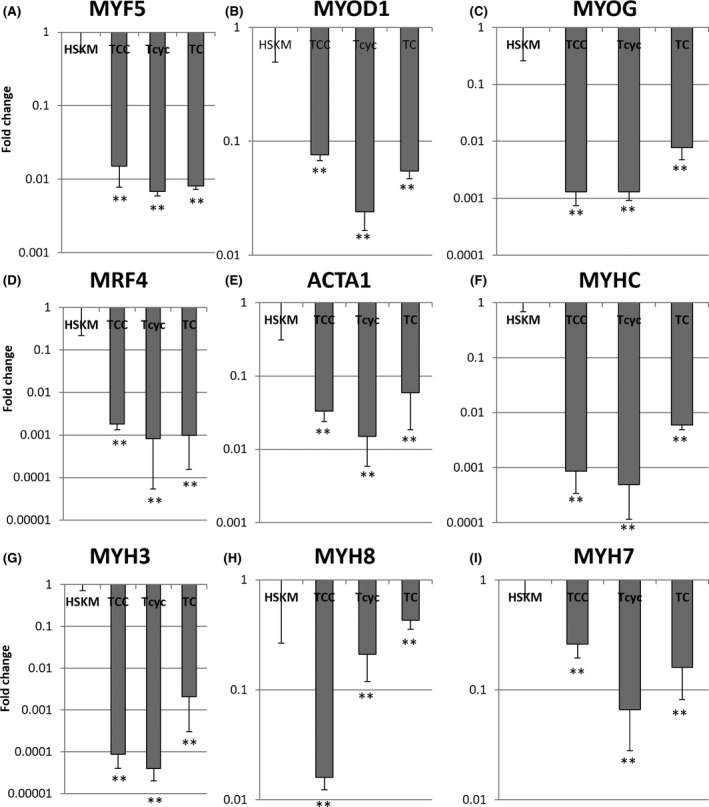
Quantitative RT‐PCR for myogenic markers in HSKM, TCC (hTERT, CDK4^R24C^, Cyclin D1), hTERT‐Cyclin D1 and hTERT‐CDK4 myoblasts that were subjected to myogenic differentiation. A, Myogenic factor 5, MYF5; B, Myogenic differentiation 1, MYOD1; C, Myogenin, MYOG; D, Myogenic factor 6 or MRF4; E, Skeletal muscle actin alpha 1, ACTA1; F, Myosin heavy chain, MYHC; G, Embryonic myosin heavy chain 3, MYH3; H, Perinatal myosin heavy chain 8, MYH8; I, Adult slow myosin heavy chain 7, MYH7. **P < 0.01, *P < 0.05, relative to HSKM

First, we turned to methods for immortalization of primary human myoblasts. While previous studies have shown that telomerase hTERT alone is insufficient to immortalize primary human myoblasts,^12^ some success has been obtained with the combination of hTERT and oncogenic CDK4^R24C^, or cyclin D1, or both.^12^, ^15^ We overexpressed lentiviral hTERT, CDK4^R24C^, and/or cyclin D1 in adult primary human skeletal muscle (hskm) myoblasts, and indeed observed that each of the combinations could effectively immortalize primary hskm myoblasts with continuously linear population doubling curves for over 100 days, whereas adult primary hskm myoblasts became senescent within 30 days and about five population doublings (Figure 1A). To assess whether the immortalized myoblasts are bona fide myoblasts, we allowed them to fuse and differentiate into multinucleated myotubes (Figure 1B). When the immortalized myoblasts were allowed to differentiate into myotubes under standard culture conditions, we found that only a small fraction of cells formed multinucleated myotubes, unlike primary myoblasts (Figure 1C‐D). The combination of hTERT, CDK4^R24C^ and cyclin D1 showed the worst differentiation potential, as determined by α‐actinin (Figure 1C) and myosin heavy chain (MHC) immunofluorescence (Figure 1D), despite manifesting the highest proliferation rate (Figure 1A). When the differentiated cells were subjected to mRNA profiling, we found that the immortalization factors severely compromised their expression of a large variety of myogenic markers, compared to primary cells (Figure 2A‐I). It can be inferred that these cells are no longer bona fide human myoblasts after immortalization by ectopic factors.

### 3.2 hESC‐myoblasts *via* embryoid body differentiation

**Figure 3:**
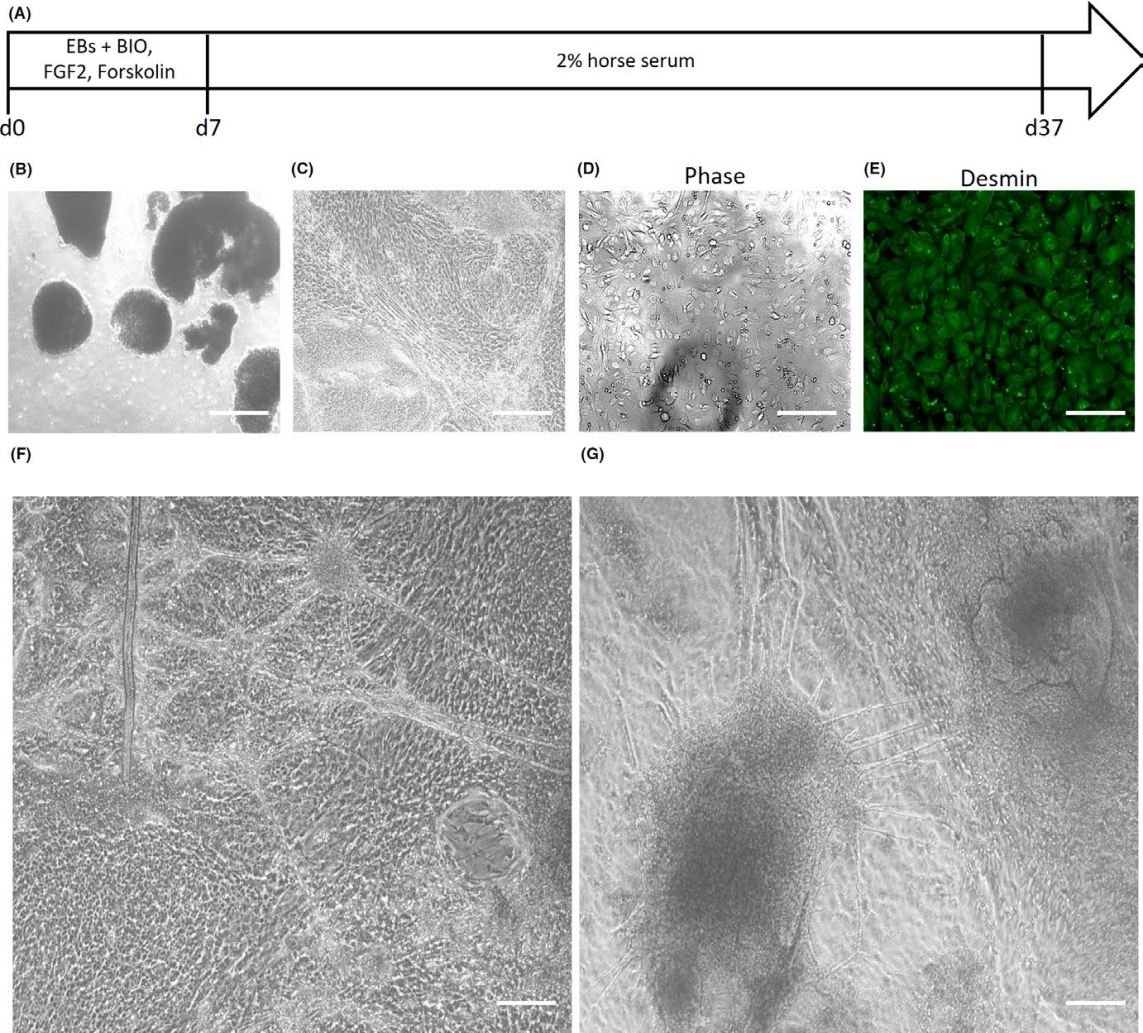
Derivation of human myoblasts and myotubes from highly proliferative human embryonic stem cells (hESCs) *via* embryoid bodies. A, Schematic of directed differentiation protocol for embryoid body (EB)‐derived myoblasts. B, Phase contrast micrographs of EBs derived from hESCs. Scale bar 200 μm. C, Phase contrast micrographs of mesodermal progenitors cultured from the hESC‐EBs. Scale bar 50 μm. D, Phase contrast micrographs, and (E) desmin immunofluorescence staining of heterogeneous myogenic cells derived from the hESC‐EB‐mesodermal progenitors. Scale bar 50 μm. F, Phase contrast micrograph of myotubes and myocytes derived from hESC‐EB‐myoblasts that were subjected to myogenic differentiation culture conditions for 2 wk. Scale bar 50 μm. G, Phase contrast micrograph of myotubes and myocytes derived from hESC‐EB‐myoblasts that were subjected to myogenic differentiation culture conditions for 3 mo. Scale bar 50 μm

Human embryonic stem cells derived from the early human embryo intrinsically possess indefinite self‐renewal capabilities, thus allowing for expansion at any scale as desired. As demonstrated before, hESCs can proliferate rapidly while preserving a stable karyotype for extended periods of time without any problems with replicative senescence.^16^ The second biggest advantage with using hESCs is that their pluripotency allows us to direct their differentiation into a variety of lineage progenitor cells. By following a previous protocol of inducing mesoderm formation *via* 3D cultures of embryoid bodies (EBs),^23^ we derived mesodermal progenitors from hESCs (Figure 3A). These EB‐derived mesodermal progenitor cells can also proliferate very rapidly, similar to their in vivo counterparts in the gastrulating embryo, thus providing yet another level of scalability in expansion and proliferation prior to the derivation of myoblasts. The problem with EB‐derived mesodermal progenitors (Figure 3B), however, is that they are highly heterogeneous (Figure 3C) and highly stochastic in their differentiation efficiencies (Figure 3D‐E). The resultant human myogenic cells only constitute 48.3 ± 3.6% of the final population, according to desmin immunofluorescence (Figure 3E). To assess whether the resultant myoblasts are bona fide myoblasts, we allowed them to fuse and differentiate into multinucleated myotubes. When the EB‐derived myoblasts were allowed to differentiate into myotubes in the presence of horse serum,^23^ we found that only a small fraction of cells formed multinucleated myotubes that emanated from a dense cluster of EB‐derived myoblasts and myocytes (Figure 3F). Most of the cells could not fuse or differentiate into myotubes even after three more months of culture (Figure 3G).

**Figure 4:**
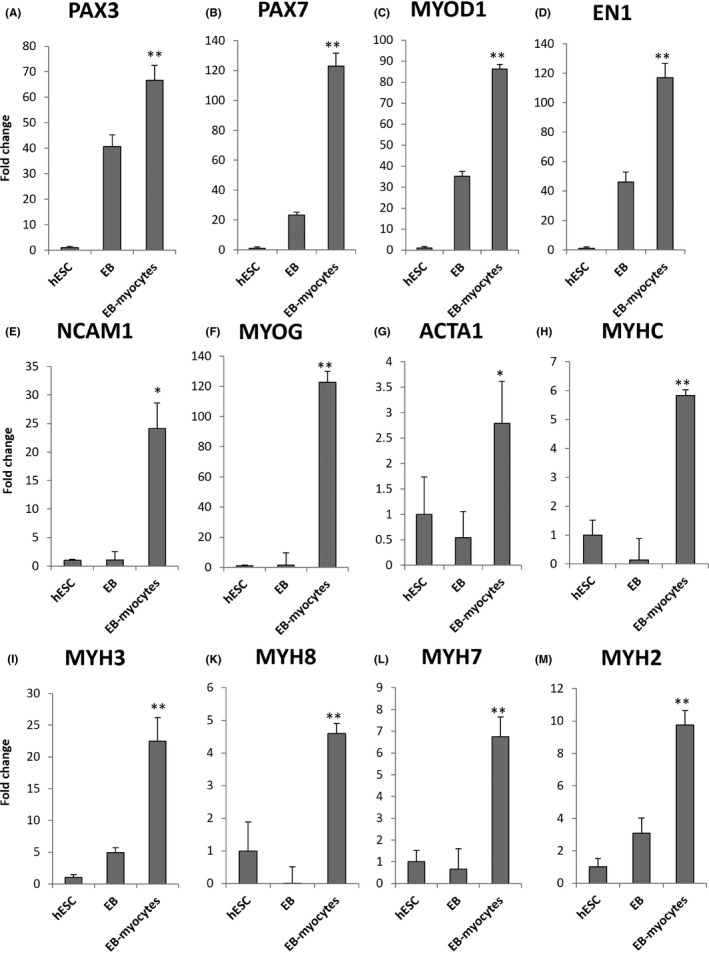
Quantitative RT‐PCR for myogenic markers in human embryonic stem cell (hESC)‐EB‐myoblasts. A, Paired box 3, PAX3; B, Paired box 7, PAX7; C, Myogenic differentiation 1, MYOD1; D, Engrailed 1, EN1; E, Neural cell adhesion molecule 1, NCAM1; F, Myogenin, MYOG; G, Skeletal muscle actin alpha 1, ACTA1; H, Myosin heavy chain, MYHC; I, Embryonic myosin heavy chain 3, MYH3; J, Perinatal myosin heavy chain 8, MYH8; K, Adult slow myosin heavy chain 7, MYH7; L, Adult fast myosin heavy chain 2, MYH2. *P < 0.05, **P < 0.01, EB‐myocytes vs EB

**Figure 5:**
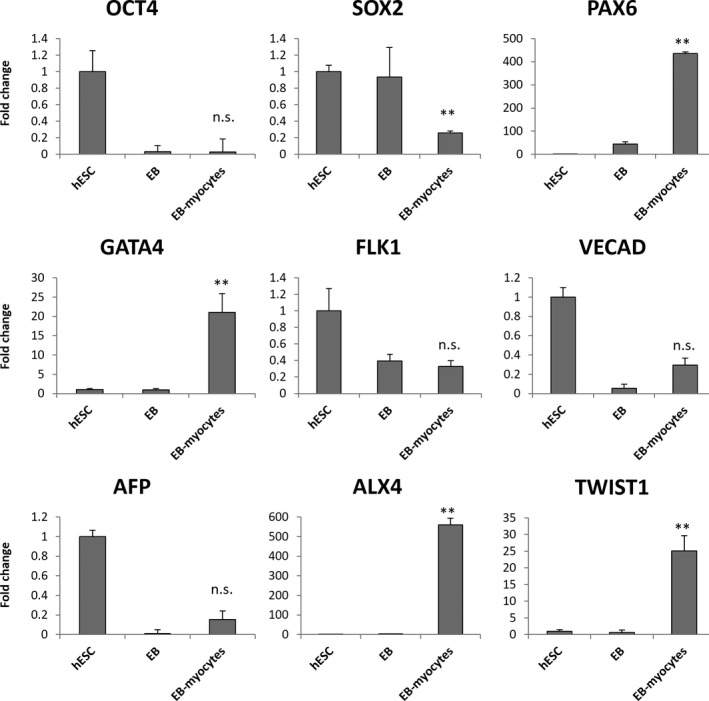
Quantitative RT‐PCR for non‐myogenic markers in human embryonic stem cell (hESC)‐EB‐myoblasts. These include the pluripotency markers OCT4 and SOX2, the neuroectoderm marker PAX6, the cardiogenic mesoderm marker GATA4, the hemangiogenic mesoderm marker FLK1, the endothelial marker VECAD, the endoderm marker AFP and the dermomyotome markers ALX4 and TWIST1. **P < 0.01, EB‐myocytes vs EB

When the embryoid bodies and EB‐derived myocytes were subjected to mRNA profiling, we found that the EBs indeed significantly upregulated their expression of myoblast markers such as PAX3, PAX7, MYOD1 and EN1 (Figure 4A‐D). Myotube markers were only specifically upregulated in EB‐derived myocytes, not EBs (Figure 4E‐L). However, the EB‐derived myocytes only showed 2‐20‐fold higher expression of myotube markers than hESCs (Figure 4E‐L). These levels of expression were significantly lower than primary human myotubes’ (Figure 7). Moreover, the EB‐derived myocytes also showed aberrant expression of the neuroectoderm marker PAX6, the cardiogenic mesoderm marker GATA4 and persistent expression of the dermomyotome markers ALX4 and TWIST1, despite several months of myogenic differentiation culture (Figure 5).

### 3.3 hESC‐myoblasts *via* mesodermal monolayer differentiation

**Figure 6:**
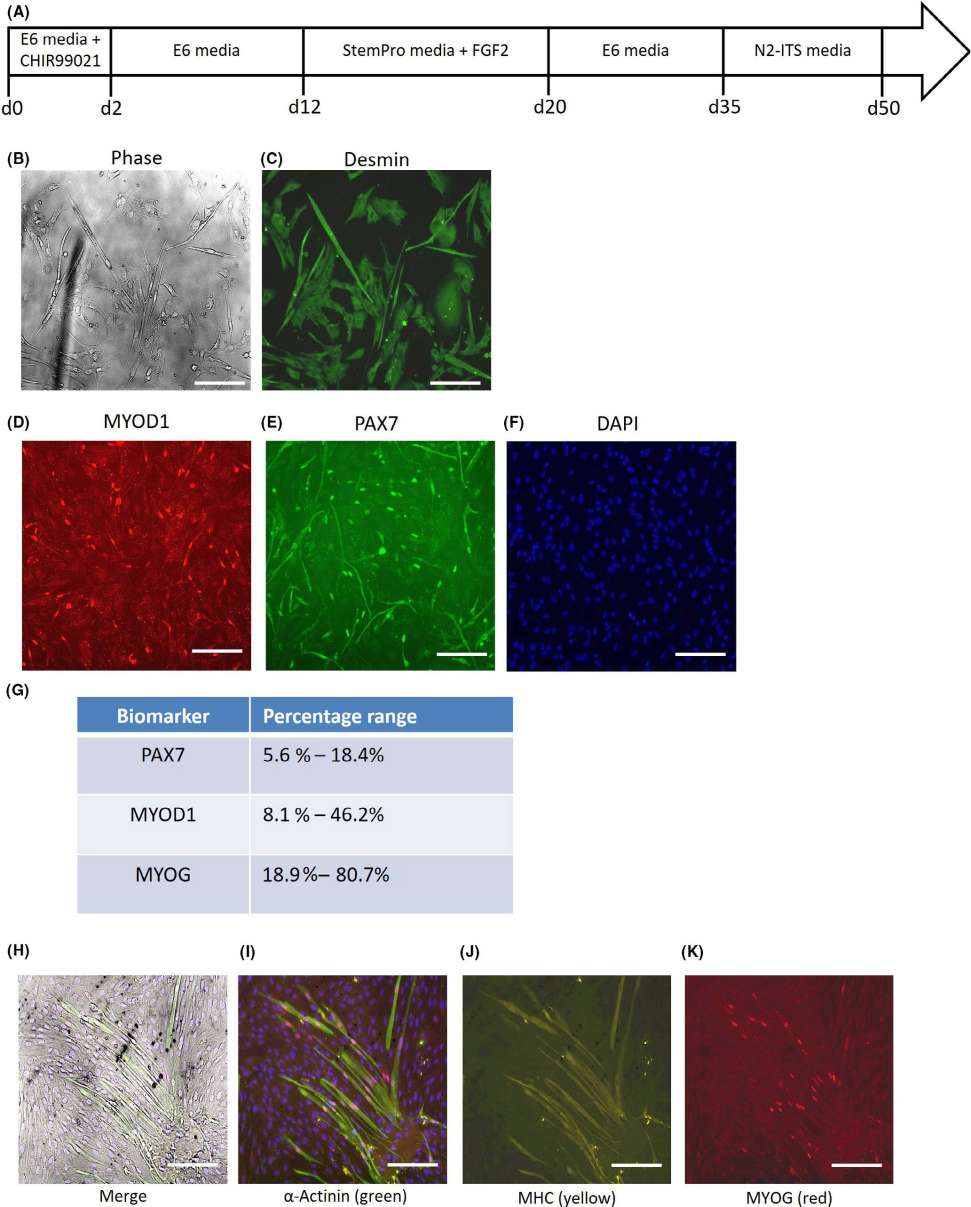
Derivation of human myoblasts and myotubes from highly proliferative human embryonic stem cells (hESCs) *via* mesodermal monolayers. A, Schematic of directed differentiation protocol for mesodermal monolayer‐derived myoblasts. B, Phase contrast micrographs, and (C) desmin immunofluorescence staining of myogenic cells derived from the hESC‐mesodermal monolayer cultures. Scale bars 50 μm. D‐F, Immunofluorescence staining of the hESC‐mesodermal monolayer‐myogenic cells for the myoblast markers (D) MYOD1 (red) and (E) PAX7 (green), with nuclei counterstained by (F) DAPI (blue). Scale bars 100 μm. G, Quantification of PAX7+, MYOD1+ and MYOG+ cells amongst the hESC‐mesodermal monolayer‐myogenic cells. H, Immunofluorescence staining for (I) α‐actinin (green), (J) myosin heavy chain (MHC, yellow) and (K) myogenin (MYOG, red) in hESC‐monolayer‐myoblasts that were subjected to myogenic differentiation culture conditions for 2 wk. Scale bars 50 μm

Based on the results above, the mesodermal monolayer method^24^, ^25^, ^26^ might produce purer myogenic cells by comparison (Figure 6A), as there are no 3D structures with stochastic sizes and variable local gradients. This proved to be true (90.6 ± 7.2% purity), according to desmin immunofluorescence (Figure 6B‐C). However, this advantage is offset by the problem of human myoblast purity, as the method produces both myoblasts and differentiating myocytes at the same time (Figure 6D‐G). Quantification by PAX7, MYOD1 and MYOG (myogenin) immunofluorescence shows that both PAX7+ myoblasts and MYOD1+ myoblasts typically only constitute a minor fraction of the population (Figure 6D‐F). The majority of the remaining cells are often MYOG+ myocytes or myotubes, although the variance can be large (Figure 6G). Thus, a significant but highly variable proportion of the final population is made up of non‐proliferative myocytes, instead of myoblasts.

When the monolayer‐derived hESC‐myoblasts were allowed to differentiate into myotubes under standard myogenic differentiation conditions,^25^, ^26^ we found that most of the cells adopted an elongated morphology typical of myocytes (Figure 6H), but only a minor fraction (21.3 ± 5.3%) of these myocytes fused and differentiated into myotubes (Figure 6H‐K). These monolayer‐derived myotubes stained positively for α‐actinin, myosin heavy chain (MHC) and nuclear myogenin (MyoG), indicating that they are terminally differentiated myotubes (Figure 6H‐K).

**Figure 7:**
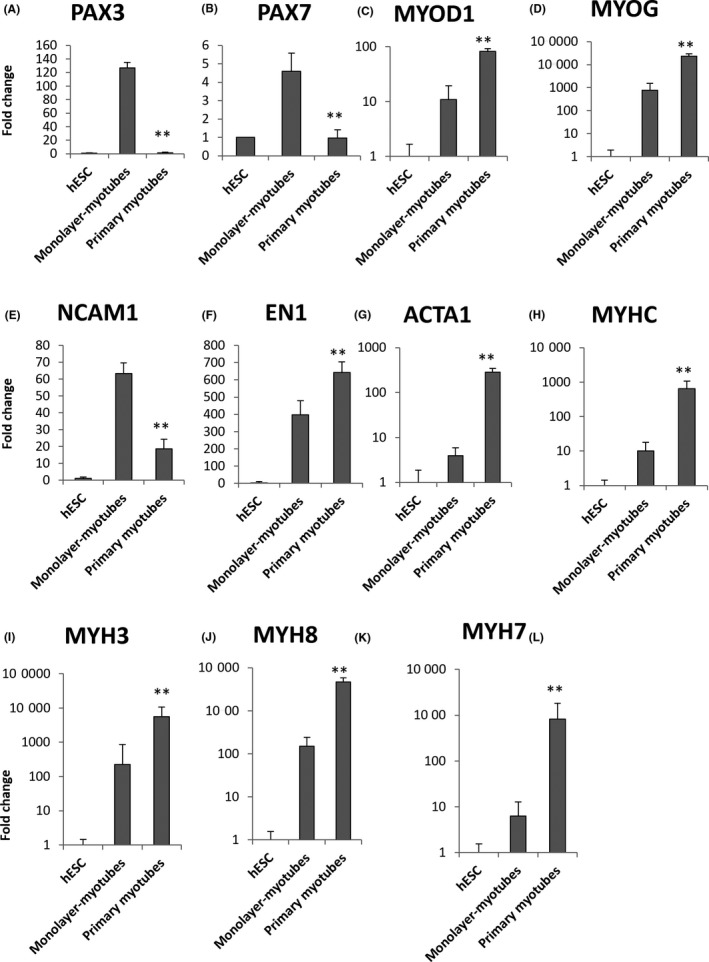
Quantitative RT‐PCR for myogenic markers in human embryonic stem cell (hESC)‐monolayer‐myotubes. A, Paired box 3, PAX3; B, Paired box 7, PAX7; C, Myogenic differentiation 1, MYOD1; D, Myogenin, MYOG; E, Neural cell adhesion molecule 1, NCAM1; F, Engrailed 1, EN1; G, Skeletal muscle actin alpha 1, ACTA1; H, Myosin heavy chain, MYHC; I, Embryonic myosin heavy chain 3, MYH3; J, Perinatal myosin heavy chain 8, MYH8; K, Adult slow myosin heavy chain 7, MYH7. **P < 0.01, Primary myotubes vs Monolayer‐myotubes

When subjected to mRNA profiling, these hESC‐monolayer‐myotubes were still expressing high levels of the paraxial mesoderm myoblast markers PAX3 and PAX7 (Figure 7A‐B), while many myogenic markers were expressed at significantly lower levels than primary human myotubes (Figure 7C‐K). This is consistent with the immunofluorescence staining, which indicates that most of the hESC‐myoblasts were still not differentiating into myotubes. One reason could be the relatively low levels of MYOD1 expression in the hESC‐monolayer myocytes (Figure 7C).

### 3.4 hESC‐myoblasts *via* MyoD overexpression

**Figure 8:**
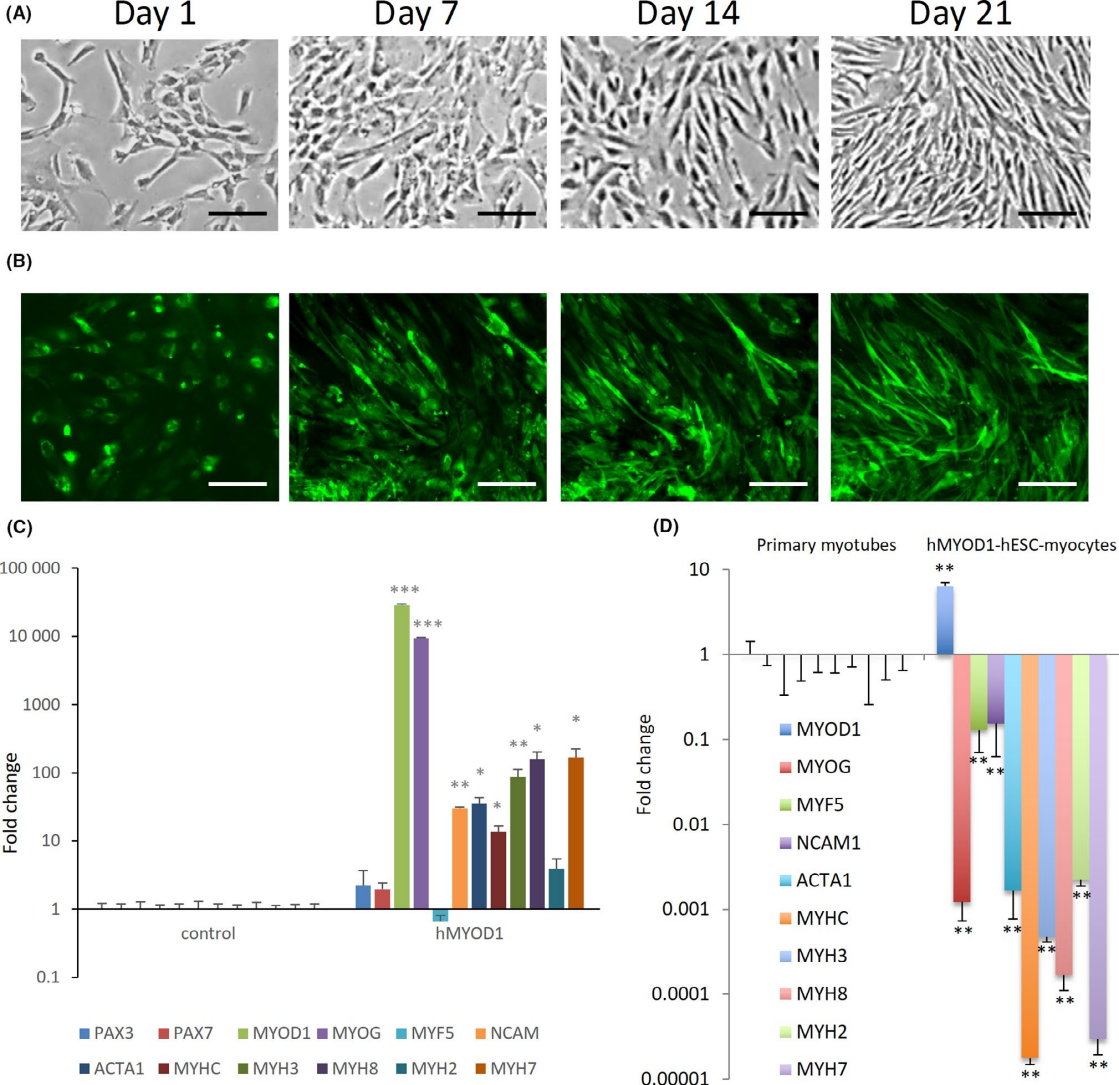
Overexpression of MyoD to derive human embryonic stem cells (hESC)‐myocytes. A, Phase contrast micrographs of hESC‐mesodermal progenitors with tamoxifen‐driven induction of human MyoD‐ER‐GFP (hMyoD1) for 1, 7, 14 and 21 d. Cells underwent elongation but no multinucleate myotubes were observed. Scale bars 20 μm. B, Localization of hMyoD1 in hESC‐mesodermal progenitors with tamoxifen‐driven induction of human MyoD‐ER‐GFP (hMyoD1) for 1, 7, 14 and 21 d. Cells underwent elongation but no multinucleate myotubes were observed. Scale bars 20 μm. C, Quantitative RT‐PCR for mRNAs of myogenic markers in hESC‐mesodermal progenitors overexpressing hMyoD1, relative to control cells. D, Quantitative RT‐PCR for mRNAs of myogenic markers in hMYOD1‐hESC‐myocytes, relative to primary myotubes. *P < 0.05, **P < 0.01, hMYOD1‐hESC‐myocytes vs controls

In an attempt to further improve the purity of the hESC‐myoblasts, and further enhance their myogenicity, we overexpressed human MYOD1 in the hESC‐mesodermal monolayer, since mouse MyoD overexpression has been widely touted to increase the myogenicity of mouse fibroblasts and stem cells.^27^, ^28^ The first thing we noticed upon tamoxifen‐induced overexpression of hMYOD1‐ER‐GFP in hESC‐mesodermal progenitors was that they rapidly adopted an elongated myocyte‐like morphology within 1 day and gradually became more homogeneous in morphology (Figure 8A). During differentiation in myotube culture conditions, the hMYOD1‐hESC‐myocytes became even more elongated over time, but they never fused into multinucleated myotubes even after 21 days of differentiation (Figure 8A‐B).

We performed mRNA profiling of the cells to ascertain their myogenic status, and found that both MYOD1 and the downstream transcription factor MYOG were significantly upregulated compared to controls (Figure 8C). Downstream myogenic biomarkers such as ACTA1, NCAM, MYH3, MYH7 and MYH8 were also significantly upregulated, suggesting that the human MYOD1 overexpression did increase myogenicity (Figure 8C). However, with the exception of MYOD1 itself, these levels of myogenic biomarker expression were still significantly lower than that of primary human myotubes (Figure 8D). Our results suggest that, unlike mouse cells, other cofactors besides MyoD are necessary to induce complete myogenesis in human mesodermal progenitor cells. It is also possible that constitutive MyoD overexpression actually inhibits downstream myogenesis in the later stages.

## 4 DISCUSSION

Rodent muscle cell lines, such as C2 or L6, have an unlimited proliferative potential and have been useful tools for the study of the cellular and molecular mechanisms involved in myogenesis. Mouse models have also been used to assess various therapeutic strategies, including MTT. However, the encouraging results obtained by grafting mouse myoblasts into the *mdx* mouse model,^5^ translated into several clinical trial failures with DMD patients.^7^, ^8^, ^9^ These clinical failures are ultimately due to intrinsic differences between mouse and human myoblasts in their proliferative capacities,^10^ and thus the scalability of human myoblasts.

While we successfully immortalized primary human myoblasts with the combined expression of CDK4^R24C^, cyclin D1 and hTERT,^12^, ^15^ resulting in rapid proliferation rates, the immortalization process severely compromised the cells’ differentiation potential. Cyclin D1 has a crucial role as a limiting factor of CDK4 kinase activity. Overexpression of cyclin D1 increases CDK4^R24C^ kinase activity to promote Rb phosphorylation, which then resulted in rapid proliferation and prevented senescence (Figure 1A). The slower proliferation of human myogenic cells immortalized by either CDK4^R24C^ or cyclin D1 alone, with hTERT, also implies that higher CDK4 activity is required for rapid proliferation. However, although the rapid proliferation program mediated by CDK4^R24C^‐cyclin D1 did overcome the senescence program, it also severely compromised the differentiation potential of the human myoblasts, likely because cell cycle inhibition is a prerequisite for proper myoblast differentiation. Moreover, these immortalized myoblasts also manifested some aberrant osteogenic and adipogenic potential.^15^ And even if the transgenes were switched off inducibly, the cells would still undergo senescence^15^ and cell death immediately (data not shown), before they can differentiate. Finally, even the hyper‐proliferative mouse muscle cell lines suffer from progressive dysfunction in survival and differentiation over time.^29^, ^30^ Taken together, these results suggest that immortalization is not likely to be a viable route for the scalable expansion of human myoblasts for clinical uses.

Inspired by the classic transdifferentiation work in mouse fibroblasts,^27^ several groups have reported the utility of using MYOD1 overexpression to obtain myogenic cells from the highly scalable hESCs and iPSCs.^22^, ^31^, ^32^, ^33^, ^34^ However, some of this work was based on patient‐specific iPSCs that are not widely available in the rest of the world, and no studies have compared them to primary human myotubes. Indeed, a previous study had shown that multiple lines of hPSCs are resistant to MYOD1‐induced myogenesis due to the absence of BAF60C.^21^ However, even with the addition of BAF60C, the resultant hMYOD1‐hPSC‐myoblasts were still far from pure, and FACS for NCAM1+ staining was necessary to further purify the hPSC‐myoblasts.^21^ This is consistent with our conclusion that other cofactors, besides MYOD1, are necessary to completely activate myogenesis in human hPSC‐myoblasts.

For clinical applications and, by inference, preclinical studies, viral transgene‐free approaches for scalable production of human myoblasts would be preferred out of safety concerns. This necessarily means that the chemically defined hPSC‐myoblasts would still be the most promising strategy. And indeed, if one judges based on the maturity of the myotubes that were obtained after myogenic differentiation, instead of overall myotube efficiency, one can also conclude that the human myoblasts derived from hPSCs are still the most promising in recapitulating their in vivo counterparts. In this regard, several groups have recently made progress in improving existing chemically defined methods to further improve the purity and maturity of the terminally differentiated human myotubes obtained from hPSCs.^35^, ^36^ Moreover, most of the extant work has been based on 2D culture on plates. Future work should also shift onto modern large‐scale microcarrier suspension cultures in bioreactors, which have been applied recently with some limited success on non‐human myoblasts.^37^, ^38^ Another dimension that deserves further exploration is the control of oxygen tension, which has been shown to exert varying effects on the proliferative capacity of myoblasts.^39^, ^40^, ^41^, ^42^. Further work would still be needed to fully optimize the chemically defined approach to produce highly pure human myoblasts and highly mature human myotubes from hPSCs in large scale.

## CONFLICT OF INTEREST

The authors declare no competing interests.

## AUTHOR CONTRIBUTIONS

M.‐W. JC, EDY, J.‐H. ET, ZJ, TBT, KY and NS‐C. analysed the data and wrote the manuscript. M.‐W. JC, EDY, J.‐H. ET, Y.‐JBC, SMCL, SLSD, ZJ, and CL performed the experiments and analysed the data.
